# Initial study of three different pathogenic microorganisms by gas chromatography-mass spectrometry

**DOI:** 10.12688/f1000research.12003.3

**Published:** 2018-01-18

**Authors:** Najmeh Karami, Fateme Mirzajani, Hassan Rezadoost, Abdollah Karimi, Fatemeh Fallah, Alireza Ghassempour, Atusa Aliahmadi

**Affiliations:** 1 Pediatric Infections Research Center, Research Institute for Children Health, Shahid Beheshti University of Medical Sciences, Tehran, Iran; 2 Department of Biotechnology, Faculty of Renewable Energies & New Technologies Engineering (NTE), Shahid Beheshti University, Tehran, Iran; 3 Department of Phytochemistry, Medicinal plants and Drugs Research Institute, Shahid Beheshti University, Tehran, Iran; 4 Department of Biology, Medicinal plants and Drugs Research Institute, Shahid Beheshti University, Tehran, Iran

**Keywords:** Candida albicans, Escherichia coli, gas chromatography-mass spectrometry, Staphylococcus aureus, volatile organic compounds

## Abstract

**Background**: Diagnoses  of  respiratory  tract  infections  usually happen  in  the  late  phase  of  the  disease  and  usually  result  in  reduction  of  the  pathogen  load after broad-spectrum  antibiotic  therapy,  but  not  in eradication of the pathogen.  The  development  of a  non-invasive,  fast,  and  accurate  method  to  detect  pathogens  has  always  been  of  interest  to  researchers  and  clinicians  alike.  Previous studies have shown that bacteria produce organic gases.  The  current  study  aimed  to  identify  the  volatile  organic  compounds  (VOCs)  produced  by three  respiratory  tract  pathogens,  including *Staphylococcus  aureus*, *Escherichia  coli * and *Candida  albicans.*

**Methods**: The  VOCs  produced  were identified by gas chromatography–mass spectrometry (GC-MS), with  prior  collection  of  microbial  volatile  compounds  using  solid  phase  microextraction  (SPME)  fiber.  The volatile compounds were collected by obtaining bacterial headspace samples.

**Results**: Results  showed  that  these  three  organisms  have  various  VOCs,  which  were  analyzed  under  different  conditions.  By ignoring common VOCs, some species-specific VOCs could be detected.  The most important VOC of *E. coli* was indole, also some important VOCs produced by *S. aureus*  were 2,3-pentandione,  cis-dihydro-α-terpinyl  acetate,  1-decyne,  1,3-heptadiene,  2,5-dimethyl  pyrazine,  ethyl  butanoate  and  cyclohexene,4-ethenyl. Furthermore,  most  of the identified  compounds  by *C.  albicans* are  alcohols.

**Conclusions**: The  detection  of  VOCs  produced  by  infectious  agents  maybe  the  key  to  make   a  rapid  and  precise  diagnosis  of  infection,  but  more  comprehensive  studies  must  be  conducted  in this  regard.

## Introduction

Infectious diseases are the main reason for morbidity and mortality in developing countries, especially among children ^1^. *Staphylococcus aureus* is a common inhabitant of the upper respiratory tract in children, and the causative agent for many infections. It is believed that people under 20 are more likely to have these bacteria. There is a greater possibility that *S. aureus* exists in the respiratory tract of infants aged 3 months or younger than in people of other ages ^2^. Moreover, *S. aureus* is colonized in the nasopharynx in 10–35% of children, and in almost 35% of the adult population ^3^.

*Escherichia coli* is one of the most significant pathogens affecting preterm infants ^4^. Some studies in developing countries have suggested that gram-negative rods (such as *E. coli*) are the major causes of infection in premature infants (0–6 days) ^5– 7^. Furthermore, infections caused by *E. coli* are one of the most important causes of death in the early neonatal period ^5^. *Candida albicans* is an opportunistic pathogen and an agent of nosocomial infection ^8^.

Generally, the causative agents of respiratory tract infections are diagnosed in late phases of the disease ^7^. Such infections need broad-spectrum antibiotic therapy, the consequences of which are a reduction in the pathogen load, but not eradication. Moreover, such therapies increase the probability of drug-resistant infections spreading ^9^. Accurate and rapid detection of pathogens is a critical step for adequate treatment of infection ^10^. and a non-invasive diagnostic method that has a high degree of accuracy needs to be developed ^11^.

It has been shown that bacteria produce organic gases. Different types of microorganisms have a distinct metabolism, and they produce various types of volatile organic compounds (VOCs) ^12– 14^. Attempts have been made to identify the VOCs of pathogenic organisms ^15– 20^. There are several sophisticated methods available that have been used for recognizing VOCs; these include gas chromatography-mass spectrometry (GC-MS) ^21^, selected ion flow tube mass spectrometry (SIFT-MS) ^22^, electronic noses (eNoses) ^23^, and ion-molecule reaction mass spectrometry (IMRMS) ^24^. Previous studies suggest that GC-MS is the most appropriate and reliable technique for the isolation and identification of VOCs ^25– 27^.

The current study aimed to identify the volatile organic compounds (VOCs) produced by three respiratory tract pathogens, including *Staphylococcus aureus*, *Escherichia coli* and *Candida albicans,* to determine if these could be used as biomarkers.

## Materials and methods

### Model organisms, medium and growth conditions

The bacterial strains used in this study were *E. coli* (ATCC 25922) and *S. aureus* (ATCC 25923), as gram-negative and gram-positive model organisms, and *C. albicans* (ATCC 10231) was used as a human pathogenic fungi model. These organisms model were obtained from the Microbiology Laboratory of Medicinal plants and Drugs Research Institute, Shahid Beheshti University. Monocultures of all strains were cultured 24 hours in nutrient agar, and then sub-cultured aerobically at 37°C in 30 ml of two different types of broth medium, Mueller Hinton broth (MB) and tryptic soy broth (TSB), in 100 ml sterilized glass bottles. For a more careful assessment of VOCs produced by each microorganism, the headspace was extracted from both media at three different time points: 2, 4 and 24 hours. To increase the possibility of VOC production, bottles containing cultured microorganism were shaken at 150 rpm during incubation time ^28^. A suspension of microorganisms with approximately OD _600_ ~0.5 in culture media was used during the headspace extraction ^10^, and the corresponding sterile broth mediums were used as the blank samples ^29^.

### Headspace extraction

A solid phase microextraction (SPME) fiber holder (57330-U, Sigma-Aldrich) containing fiber coated with divinyl benzene/carboxen/poly dimethyl siloxane 50/30 µm (DVB/CAR/PDMS) (57328-U, Sigma-Aldrich) was used for absorption of volatile compounds from the headspace of pathogens. To provide conditions that increase the rate of VOC absorption, after incubation time, 2ml of NaCl 36% was added to each culture. Then the DVB/CAR/PDMS fiber was suspended from the top of the bottle containing the culture and placed on a magnetic stirrer hotplate at 70°C for 30 minutes ^30^. After that, the fiber was placed at the injection site of GC-MS and all the absorbed VOCs entered the device. Eventually each VOC is represented as a chromatogram peak in the monitor that is connected to the GC-MS. For thermal desorption, the SPME fiber remained in the injector for 2 minutes before it was exposed to the headspace of the pathogen samples ^31^. To avoid possible false discoveries each state was tested at least three times.

### GC-MS

To study the bacterial VOCs, a Thermo-Finnigan Trace GC-MS system (Thermo Quest-Finnigan Co) equipped with a DB-5 column (60 m length, 0.25 mm inner diameter, and 0.25 μm film thickness) with helium carrier gas at a flow rate of 1.1 ml/min was used. The starting temperature was 50°C, increasing at a rate of 10°C/minute up to 250°C. The GC-MS was set in splitless mode and a quadrupole ion trap with ionization energy of 70 eV was used in the filament.

VOCs were identified using the National Institute of Standards and Technology (NIST) reference library. To analyze the GC-MS data, Xcalibur 3.0 with Foundation 3.0 SP2 software (Thermo Fisher Scientific) was used, and the kovats retention index (RI) was calculated for each chromatographic peak.

When calculating the RI, a series of standards were used: n-alkanes were injected into the GC-MS the day before starting experiments, using the same temperature profile that would be used for the analysis of VOCs. The NIST17 Mass Spectral Library (NIST7/2017/EPA/NIH) was used to identify each compound according to its RI. Since there may be several types of volatile compounds have similar RI, to validate the final results extensive studies were also performed by a phytochemist to determine if the compounds were organic. The common VOCs released from the sterile environment (Blank samples) and tests were not considered.

## Results

The VOCs produced by *S. aureus*, *E. coli* and *C. albicans* were assessed under six different conditions (using two types of media and taking measurements at three time points). The Xcalibur raw files for these three pathogens are available at https://doi.org/10.6084/m9.figshare.5178004.v1 ^32^.

**Figure 1.**
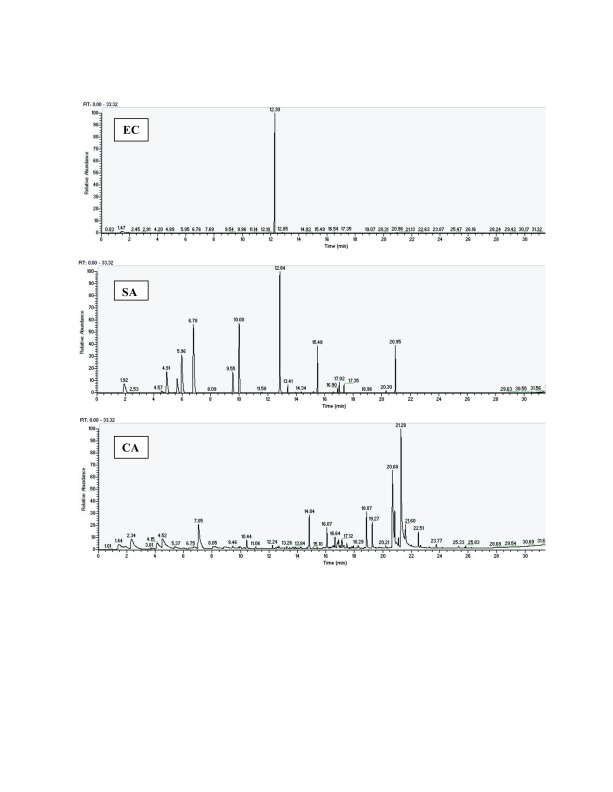
Three chromatograms, for samples taken 4 hours after culture in TSB media. EC: E. coli, SA: *S. aureus* and CA: *C. albicans*. The other chromatograms are available in the Supplementary material.

One chromatogram of the six chromatograms obtained is displayed in **Figure 1, showing the chromatogram obtained** 4 hours after culture in TSB medium, for each pathogen. The five other chromatograms are also available, as Supplementary File S1, Supplementary File S2, Supplementary File S3, Supplementary File S4 and Supplementary File S5.

The processed GC-MS data obtained in the current study is available in a total of 18 tables as supplementary GC-MS data. It shows the details of the VOCs detected for each of the three pathogens, each analyzed under different conditions (using two types of media and taking measurements at three time points, as explained above).

For a better overview the detected VOCs are shown in three tables (at the 2 hour time point in Table 1, at the 4 hour time point in Table 2 and at the 24 hour time point in Table 3), alongside the percentage of the total area that the average peak of the detected VOC covered. In other words it is proportional to amount of the compound that is present.

**Table 1.** The identified VOCs for *E. coli*, *S. aureus* and *C. albicans,* and the percentage of the total area that their average peak covered (peak area %), after 2hours in MB and TSB media. In total, 25 types of VOCs by *E. coli*, 33 types by *S. aureus* and 28 types by *C. albicans* were generated in this period.

Compound	*E. coli* in MB	*E. coli* in TSB	*S. aureus* in MB	*S. aureus* in TSB	*C. albicans* in MB	*C. albicans* in TSB
(e)-2-hexyl ester-butanoic acid	1.84	0.79	-	-	6.75	3.78
1-(1,5-dimethyl-4-hexyl-4-methyl-benzene	3.19	0.41	-	-	-	-
1,2-benzenedicarboxylic acid	-	-	0.39	-	-	0.2
1,2-butadiene	-	-	-	1.73	-	-
1,3-butadiene	-	-	-	-	-	26.68
1,3-heptadiene	-	-	-	4.88	-	4.55
1,5-decadiene	-	-	-	-	-	0.86
1,9-decadiene	0.05	0.05	-	-	-	0.39
1-decyne	-	0.07	0.85	-	1.55	1.55
1-penten-3-ol	-	0.02	-	5.14	-	-
2,3-pentandione	-	1.33	-	-	-	-
2,5-(1,1-dimethylethyl)-phenol	0.13	0.1	-	0.11	-	0.43
2,5-dimethyl pyrazine	-	-	-	20.19	-	3.07
2,6-bis(1,1-dimethylethyl)-4-methyl-phenol	-	0.04	0.5	-	-	0.64
2,6-dibutyl-2,5-cyclohexadiene-1,4-dione	0.03	-	-	-	-	-
2-ethenyl-6-methyl-pyrazine	1.1	0.63	6.58	6.63	-	3.63
2-ethyl hexanol	-	-	-	2.32	-	-
2-heptanone	0.05	-	-	2.31	-	-
2-hexan-1-ol	-	-	-	-	-	0.22
2-methyl-2-undecanethiol	0.24	0.13	1.98	1.09	-	-
3-methyl-1,5-heptadiene	-	-	-	-	3.77	1.03
3-propionyloxypentadecane	0.57	0.18	7.36	1.3	2	0.61
4-t-butyl-2-(1-methyl-2- nitroethyl)cyclohexane	0.93	0.73	12.17	5.4	10.98	2.45
5.5-dodecadinyl-1, 12-diol	-	-	0.48	0.5	1.19	6.38
allyl butylhydroquinone	-	-	-	0.31	-	-
anisol	-	0.05	-	1.19	-	-
benzaldehyde	2.13	1.34	3.22	8.98	-	0.64
benzene acetaldehyde	-	-	8.74	7.04	-	-
benzophenone	0.03	-	-	-	-	-
bisabolene	1.21	0.03	-	-	-	-
butyl cyclohexyl acetate	-	-	-	0.4	-	-
butyraldehyde	-	-	-	-	-	0.67
cadinene	-	-	-	-	-	1.79
carbamic acid	-	-	-	-	48.49	0.5
caryophyllene	-	-	-	0.09	-	-
cedran-1,8-diol	0.14	0.09	2.63	0.39	-	0.48
cedrol	-	-	0.71	0.23	-	-
copaene	0.01	-	-	-	-	-
cyclohexene, 4-ethenyl-	-	-	29.64	2.18	-	0.47
decanol	-	0.93	-	-	-	-
decene	-	-	-	2.85	-	-
dimethyl octenal	-	-	-	1.39	-	-
dimethyl ethyl cyclohexanol	-	-	-	1.04	-	0.59
dodecane	0.06	-	-	0.96	-	-
dodecanol	-	0.27	-	-	-	-
dodecenol	-	-	-	-	-	0.5
eicosane	-	-	-	-	-	0.12
ethyl butanoate	-	-	-	-	5.31	4.64
heptadecane	-	-	12.35	5.33	-	-
humulen	-	-	0.71	-	-	-
indole	82.61	90.97	-	0.48	-	-
limonene	0.68	-	-	-	-	-
longifolene	-	-	-	-	4.96	0.43
longifolene	-	-	-	0.52	-	-
methone	-	-	-	7.49	-	1.4
muurola-4,5-diene	0.47	-	-	-	-	-
naphthalenol	-	0.84	-	0.21	-	0.44
neryl acetate	0.06	0.03	-	-	-	-
nonadecanone	-	-	0.62	-	-	-
ocimene	-	-	-	-	-	2.24
octacosane	0.41	0.06	1.37	1.22	1.2	1.02
octyl acetate	-	-	-	-	-	0.4
pentadecane	0.03	-	0.86	0.68	-	4.1
phthalic acid, butyl ester	0.19	0.13	0.97	-	-	0.22
phenyl ethyl pyrrole	-	0.06	-	-	-	-
sesquiphellandrene	1.1	-	-	-	-	4.79
tetra butyl cyclohexyl acetate	-	-	1.7	-	-	-
tetradecane	0.01	-	-	-	-	-
tetradecanol	-	-	-	-	-	0.34
zingiberene	1.63	-	1.03	-	2.87	7.91
α-acetoxydihydrocoumarin	-	0.52	1.88	0.25	-	-
β-santalol	-	-	-	-	4.87	1.34

**Table 2.** The identified VOCs for *E. coli*, *S. aureus* and *C. albicans,* and the percentage of the total area that their average peak covered (peak area %), after 4 hours in MB and TSB media. In total, 9 types of VOCs by E. coli, 19 types by *S. aureus* and 42 types by *C. albicans* were generated in this period.

Compound	*E. coli* in MB	*E. coli* in TSB	*S. aureus* in MB	*S. aureus* in TSB	*C. albicans* in MB	*C. albicans* in TSB
(e)-2-hexyl ester-butanoic acid	-	-	-	-	6.64	4.24
(z)-2-octene-1-ol	-	-	-	-	0.69	0.54
(z)-4-decan-1-ol	-	-	-	-	0.70	0.46
1,2-benzenedicarboxylic acid	-	-	-	-	0.29	0.31
1,2-butadiene	-	-	0.37	4.02	-	-
1,3-butadiene	-	-	-	-	1.40	-
1,3-heptadiene	-	-	81.33	-	0.23	0.47
1,5-decadiene	-	-	-	-	1.01	3.00
1,9-decadiene	-	0.01	-	-	0.20	3.59
1-decyne	2.36	-	0.59	16.47	2.81	1.40
1-methoxy-2-propanol	-	-	0.20	-	-	-
2-(phenylmethylene)-octanal	-	-	-	-	1.55	1.63
2,3-pentandione	-	-	7.07	21.67	-	-
2,5-(1,1-dimethylethyl)-phenol	-	-	-	-	0.69	0.43
2,5-dimethyl pyrazine	-	-	-	-	-	3.48
2-acetyl-1-pyrroline	-	0.07	-	-	-	-
2-ethenyl-6-methyl-pyrazine	0.01	-	0.80	-	7.33	-
2-ethyl hexanol	-	-	-	1.05	-	-
2-heptanone	0.02	0.33	-	-	-	-
2-hexan-1-ol	-	-	-	-	0.33	0.23
2h-tetrazole-5-carboxylicacid, 2-phenyl	-	-	0.48	-	1.44	1.59
2-methyl-2-undecanethiol	-	-	0.24	-	-	-
3-methyl-1,5-heptadiene	-	-	0.60	-	0.76	0.53
4-t-butyl-2-(1-methyl-2- nitroethyl)cyclohexane	-	-	-	-	6.05	5.81
5.5-dodecadinyl-1, 12-diol	-	-	-	-	5.16	0.51
6-methyl-5-hepten-2-one	-	-	-	-	0.31	0.20
benzaldehyde	-	-	-	-	0.83	0.68
butyraldehyde	-	-	0.38	-	-	-
cadinene	-	-	-	-	0.79	0.34
carbamic acid	-	-	-	-	18.10	8.19
caryophyllene	-	0.02	-	4.88	-	-
cedrol	-	-	-	-	0.36	0.36
cis-dihydro-α-terpinyl acetate	-	-	-	17.90	-	-
cyclohexene, 4-ethenyl-	-	-	0.08	6.77	-	-
dimethyl octenal	-	-	-	-	0.58	-
dimethylethyl cyclohexanol	-	-	-	-	0.79	0.22
dodecenal	-	-	-	-	0.52	0.39
dodecenol	-	-	-	-	0.61	2.54
eicosane	-	-	-	-	-	0.29
ethyl butanoate	0.01	-	7.57	12.21	6.63	0.73
indole	97.05	99.46	-	0.82	0.34	-
levomenthol	-	-	-	3.74	-	-
longifolene	-	-	-	-	0.31	0.26
longifolol	-	-	-	-	5.66	22.07
methyl isopropyl hexenal	-	-	-	-	1.11	0.59
naphthalenol	-	-	-	-	-	0.37
octacosane	-	-	-	-	1.74	1.60
octyl acetate	-	-	-	-	0.34	-
pentadecane	-	-	-	0.87	1.80	1.38
phthalic acid, butyl ester	-	-	-	-	0.60	0.36
tetradecanol	-	-	-	-	0.61	0.26
tridecanol	-	-	-	-	0.34	0.34
zingiberene	-	-	-	-	1.27	1.12
β-santalol	-	0.03	-	5.16	6.52	14.15
sesquiphellandrene	-	-	-	-	2.00	0.70

**Table 3.** The identified VOCs for *E. coli*, *S. aureus* and *C. albicans,* and the percentage of the total area that their average peak covered (peak area %), after 24 hours in MB and TSB media. In total, 16 types of VOCs by *E. coli*, 26 types by *S. aureus* and 27 types by *C. albicans* were generated in this period.

Compound	*E. coli* in MB	*E. coli* in TSB	*S. aureus* in MB	*S. aureus* in TSB	*C. albicans* in MB	*C. albicans* in TSB
(e)-2-hexyl ester-butanoic acid	0.04	0.03	0.21	0.04	0.50	-
(z)-2-octene-1-ol	-	-	-	-	-	0.18
(z)-4-decan-1-ol	-	-	-	-	-	0.60
1,2-benzenedicarboxulic acid	-	-	-	-	0.13	0.28
1,2-butadiene	-	-	3.71	0.10	-	-
1,3-heptadiene	-	-	21.75	-	-	-
1,5-decadiene	-	-	-	-	0.10	0.26
1,9-decadiene	0.02	0.03	-	-	-	-
1-decyne	-	0.02	0.81	59.78	-	0.75
1-methoxy-2-propanol	-	-	6.74	0.02	-	-
2-(phenylmethylene)-octanal	-	-	-	-	0.71	0.86
2,3-pentandione	0.03	0.48	15.53	0.66	-	-
2,5-dimethyl pyrazine	-	-	0.62	-	-	0.55
2-acetyl-1-pyrroline	-	6.37	-	1.11	-	-
2-decenal	-	-	0.06	0.01	-	-
2-ethenyl-6-methyl-pyrazine	0.06	-	1.35	-	0.39	-
2-ethyl hexanol	-	-	0.13	0.02	-	0.51
2-heptanone	-	0.21	-	-	-	-
2h-tetrazole-5-carboxylicacid, 2-phenyl	-	-	3.21	-	-	-
2-methyl tetradecane	0.05	0.03	-	-	-	-
2-methyl-1-propanol	-	-	0.15	0.04	5.89	16.03
2-methyl-2-undecanethiol	0.10	-	2.54	-	-	61.65
2-octyl-1-ol	-	-	-	-	0.15	-
2-octyne	-	-	-	-	-	0.27
3-methy-4-pentene-3-ol	-	-	-	-	-	0.13
3-methyl-1,5-heptadiene	-	-	0.19	-	-	-
3-methyl-1-pentene	-	-	-	-	-	0.41
-4-t-butyl-2-(1-methyl-2- nitroethyl)cyclohexane	-	-	-	-	80.87	
5.5-dodecadinyl-1, 12-diol	0.01	0.03	0.77	0.05	-	-
butyraldehyde	-	-	1.41	0.09	0.14	0.24
carbamic acid	-	-	-	-	0.48	-
caryophyllene	-	0.21	-	0.14	-	1.25
cedrol	-	-	-	-	1.93	2.68
cis-dihydro-α-terpinyl acetate	-	0.64	-	34.77	-	-
cyclohexene, 4-ethenyl-	-	-	3.54	0.15	-	-
ethyl acetoacetate	-	-	-	-	-	0.46
ethyl butanoate	0.06	0.31	28.72	0.46	-	1.64
indole	99.61	88.86	0.07	0.02	-	-
levomenthol	-	-	-	1.66	-	-
longifolol	-	-	-	-	0.33	0.24
octacosane	-	-	-	-	0.33	0.44
pentadecane	-	0.35	0.17	0.08	-	-
thiophene	-	-	0.18	-	-	-
zingiberene	-	-	-	-	0.20	-
β-santalol	-	0.49	-	0.21	-	1.21

Some VOCs were common among organisms and were generated by two or three organisms at an approximately equal rate, including 1,2-benzenedicarboxylic acid, 1,9-decadiene, 2,5-(1,1-dimethylethyl)-phenol, 2,6-bis(1,1-dimethylethyl)-4-methyl-phenol, 3-propionyl oxy pentadecane and anisol ( Table 1). Some common VOCs were produced at a greater rate between one organism and another. It can be concluded that these VOCs could also be more important in the organism that produces greater quantities. 1-penten-3-ol was produced from *E . coli* in TSB medium after 2 hours (0.02%); under identical conditions, more of it was produced by *S. aureus* (5.14%) than by *E. coli.* Furthermore, indole was produced from E. coli after 2 hours of culture in two types of medium (82.61% for MB and 90.97% for TSB) and was also produced by S. aureus after 2 hours in TSB medium, although at a much lower rate (0.48%) ( Table 1).

Uncommon VOCs of *E. coli* detected 2 hours after culture included 1-(1,5-dimethyl)-4-hexyl-4-methyl-benzene, 2,3-pentandione, 2,6-dibutyl-2,5-cyclohexadiene-1,4-dione, benzophenone, bisabolene, copaene, decanol, dodecanol, indole, limonene, muurola-4,5-diene, neryl acetate, phenyl ethyl pyrrole and tetradecane ( Table 1).

Uncommon VOCs of *S. aureus* detected 2 hours after culture included 1,2-butadiene, 1-penten-3-ol, 2,5-dimethyl pyrazine, 2-ethyl hexanol, allyl butyl hydroquinone, benzene acetaldehyde, butyl cyclohexyl acetate, caryophyllene, cedrol, cyclohexene, 4-ethenyl-, decene, dimethyl octenal, heptadecane, humulen, longifolene, methone, nonadecanone and tetrabutyl cyclohexyl acetate ( Table 1).

Uncommon VOCs of *C. albicans* detected 2 hours after culture included 1,3-butadiene, 1,5-decadiene, 2-hexan-1-ol, 3-methyl-1,5-heptadiene, butyraldehyde, cadinene, carbamic acid, dodecenol, eicosane, ethyl butanoate, longifolene, ocimene, octyl acetate, tetradecanol and β-santaloland ( Table 1).

Uncommon VOCs of *E. coli* identified 4 hours after culture included 1,9-decadiene, 2-acetyl-1-pyrroline, 2-heptanone and indole ( Table 2).

Uncommon VOCs of *S. aureus* identified 4 hours after culture included 1,2-butadiene, 1,3-heptadiene, 1-decyne, 1-methoxy-2-propanol, 2,3-pentandione, 2-ethyl hexanol, 2-methyl-2-undecanethiol, butyraldehyde, cis-dihydro-α-terpinyl acetate, cyclohexene,4-ethenyl- and levomenthol ( Table 2).

Uncommon VOCs of *C. albicans* identified 4 hours after culture included (e)-2-hexyl ester- butanoic acid, (z)-2-octene-1-ol, (z)-4-decan-1-ol, 1,2-benzenedicarboxulic acid, 1,3-butadiene, 1,5-decadiene, 2-(phenyl methylene)-octanal, 2,5-(1,1-dimethylethyl)-phenol, 2,5-dimethyl pyrazine, 2-ethenyl-6-methyl-pyrazine, 2-hexan-1-ol, 4-t-butyl-2-(1-methyl-2-nitroethyl) cyclohexane, 5.5-dodecadinyl-1, 12-diol, 6-methyl-5-hepten-2-one, benzaldehyde, cadinene, carbamic acid, cedrol, , dimethyl octenal, dimethyl ethyl cyclohexanol, dodecenal, dodecenol, eicosane, longifolene, longifolol, methyl isopropyl hexenal, naphthalenol, octacosane, octyl acetate, phthalic acid butyl ester, tetradecanol, tridecanol, zingiberene and sesquiphellandrene ( Table 2).

Uncommon VOCs of *E. coli* identified 24 hours after culture included 1,9-decadiene, 2-acetyl-1-pyrroline, 2-heptanone, 2-methyl tetradecane and indole ( Table 3).

Uncommon VOCs of *S. aureus* identified in 24 hours after culture were included; 1,2-butadiene, 1,3-heptadiene, 1-decyne, 1-methoxy-2-propanol, 2,3-pentandione, 2,5-dimethyl pyrazine, 2-decenal, 2h-tetrazole-5-carboxylicacid, 2-phenyl, 3-methyl-1,5-heptadiene, caryophyllene, cis-dihydro-α-terpinyl acetate, cyclohexene,4-ethenyl-, ethyl butanoate, levomenthol and thiophene ( Table 3).

Uncommon VOCs of *C. albicans* identified 24 hours after culture included (z)-2-octene-1-ol, (z)-4-decan-1-ol, 1,2-benzenedicarboxylic acid, 1,5-decadiene, 2-(phenyl methylene)-octanal, 2,5-dimethyl pyrazine, 2-methyl-1-propanol, 2-methyl-2-undecanethiol, 2-octyl-1-ol, 2-octyne, 3-methy-4-pentene-3-ol, 3-methyl-1-pentene, 4-t-butyl-2-(1-methyl-2-nitroethyl) cyclohexane, carbamic acid, cedrol, , ethyl acetoacetate, longifolol, octacosane and zingiberene ( Table 3).

## Discussion

As previous studies have shown, organisms are able to produce either common or specific VOCs ^33– 35^. In the current study, GC-MS was used to detect VOCs generated by three pathogenic organisms in the human respiratory tract. The VOCs of *E. coli*, *S. aureus* and *C. albicans* were analyzed at three different time points, using two different types of media ( Figure 1).

Results of the current study suggest that VOCs exclusively produced by *E. coli* are 1-(1,5-dimethyl)-4-hexyl-4-methyl-benzene, 2,6-dibutyl-2,5-cyclohexadiene-1,4-dione, benzophenone, bisabolene, copaene, decanol, dodecanol, indole, limonene, muurola-4,5-diene, nerylacetate, phenyl ethyl pyrrole, sesquiphellandrene, tetradecane, 2-acetyl-1-pyrroline and 2-methyl tetradecane. The most important compound among these is Indole, because it is generated at the three time points and also it was the most produced VOC by *E. coli* (at least 82%). Other studies have confirmed this finding ^28, 29, 35^. *E. coli* produced tryptophanase and this enzyme degrades tryptophan to indole and the other compounds ^36^. In future studies, it is advisable to measure the amount of indole in the exhaled air of infected patients with *E. coli* and compare it with the current results. This is because in the patient’s lungs the level of tryptophan is not the same as culture medium. It is also suggested that the amount of released indole from this bacterium should be evaluated under at *in-vitro* conditions and with using the simplest culture medium (relative to TSB and MB). In this way, we will have a more detailed thought of the importance of the Indole production by *E. coli*.

The current study has shown that the specific VOCs produced by *S. aureus* are 1,2-butadiene, 1-penten-3-ol, 2,5-dimethyl pyrazine, 2-ethyl hexanol, allyl butyl hydroquinone, benzene acetaldehyde, butylcyclohexyl acetate, caryophyllene, cyclohexene, 4-ethenyl-, decene, heptadecane, humulen, longifolene, methone, nonadecanone, tetrabutylcyclohexyl acetate, 1,3-heptadiene, 1-decyne, 1-methoxy-2-propanol, 2,3-pentandione, cis-dihydro-α-terpinyl acetate, levomenthol, 2-decenal, ethyl butanoate and thiophene. Moreover, 1,2-butadiene, 2,5-dimethyl pyrazine, 2-ethyl hexanol, caryophyllene, cyclohexene, 4-ethenyl, 1,3-heptadiene, 1-decyne, 1-methoxy-2-propanol, 2,3-pentandione, cis-dihydro-α-terpinyl acetate, and levomenthol were detected under more than one of the six conditions that were tested, so they are significant. Another important point is that the percentage of the total area that the average peaks for 2,3-pentandione, cis-dihydro-α-terpinyl acetate, 1-decyne, 1,3-heptadiene, 2,5-dimethyl pyrazine, ethyl butanoate and cyclohexene,4-ethenyl covered were at least 15%; thus, they are remarkable VOCs for *S. aureus.* Some of the VOCs produced by *S. aureus* in the current study have been reported in other studies ^34, 37^ but some of them have not ^28, 33^. The origin of all produced VOCs is not exactly known. However it is believed some released VOCs by this bacterium is because of the ability to degrade amino acids in its growth environment ^11^.

This study suggested that the specific VOCs produced by *C. albicans* include 1,3-butadiene, 1,5-decadiene, 2-hexan-1-ol, cadinene, carbamic acid, dodecenol, eicosane, longifolene, ocimene, octyl acetate, tetradecanol, β-sesquiphellandrene, (z)-2-octene-1-ol, (z)-4-decan-1-ol, 2-(phenyl methylene)-octanal, 4-t-butyl-2-(1-methyl-2-nitroethyl) cyclohexane, longifolol, 6-methyl-5-hepten-2-one, dodecenal, methyl isopropyl hexenal, tridecanol, 2-methyl-2-undecanethiol, 2-octyl-1-ol, 2-octyne, 3-methy-4-pentene-3-ol, 2-methyl-1-propanol and 3-methyl-1-pentene. also, 1,3-butadiene, 1,5-decadiene, 2-hexan-1-ol, cadinene, carbamic acid, dodecenol, eicosane, longifolene, octyl acetate, tetradecanol, β-sesquiphellandrene, (z)-2-octene-1-ol, (z)-4-decan-1-ol, 2-(phenyl methylene)-octanal, 4-t-butyl-2-(1-methyl-2-nitroethyl) cyclohexane, longifolol, octyl acetate, β-sesquiphellandreneand 2-methyl-2-undecanethiol were detected under more than one of the six conditions that were tested, so they are significant. Furthermore, 1,3-butadiene, carbamic acid, longifolol, β-santalol, 2-methyl-1-propanol, 2-methyl-2-undecanethiol and 4-t-butyl-2-(1-methyl-2-nitroethyl) cyclohexane were produced in greater quantities . Several studies have analyzed the VOCs of *C. albicans* and have noted that most of these identified compounds are alcohols ^38– 40^. That is because if favorable growth conditions are available for this bacterium (a sufficient level of oxygen, aromatic amino acids, and an alkaline pH) will produce large amounts of alcohol that results from its metabolism ^41^.

It is suggested that the findings of future studies on the exhaust air of respiratory infections patients with these three pathogens should be compared with the identified VOCs in this study. Although there may be some differences between the results of *in-vitro* and *in-vivo* studies there seems to be significant similarities over the dominant detected VOCs.

Finding a non-invasive and rapid method for diagnosis of infectious agents is a subject of interest, so it has been investigated in several studies ^33, 42– 45^. The current study showed that using SPME fiber and GC-MS for extraction and detection of VOCs allowed detection of more specific VOCs for the three pathogenic respiratory tract organisms, *E. coli*, *S. aureus* and *C. albicans*, which could be used as biomarkers for their identification. It is essential that more comprehensive studies be conducted to create a more complete profile of VOCs for these organisms, and so that the methods can be developed further.

## Data availability

The Xcalibur raw files for the three studied pathogens are available at https://doi.org/10.6084/m9.figshare.5178004.v1 ^32^.

## Supplementary material

**Supplementary File S1:** Three chromatograms, for samples taken 2 hours after culture in MB media. EC: E. coli, SA: *S. aureus* and CA: *C. albicans*.

**Supplementary File S2:** Three chromatograms, for samples taken 4 hours after culture in MB media. EC: E. coli, SA: *S. aureus* and CA: *C. albicans*.

**Supplementary File S3:** Three chromatograms, for samples taken 24 hours after culture in MB media. EC: E. coli, SA: *S. aureus* and CA: *C. albicans*.

**Supplementary File S4:** Three chromatograms, for samples taken 2 hours after culture in TSB media. EC: *E. coli*, SA: *S. aureus* and CA: *C. albicans*.

**Supplementary File S5:** Three chromatograms, for samples taken 24 hours after culture in TSB media. EC: *E. coli*, SA: *S. aureus* and CA: *C. albicans*.

**Supplementary File S6:** GC-MS data analysis, showing the details of the detected VOCs of three pathogens in 6 modes.
