# Supplementary figures and images for: Initial study of three different pathogenic microorganisms by gas chromatography-mass spectrometry
Source: F1000Res. 2018 Jan 18;6:1415. Originally published 2017 Aug 10. [Version 3] doi: 10.12688/f1000research.12003.3 (PMC5760968; doi:10.12688/f1000research.12003.3)

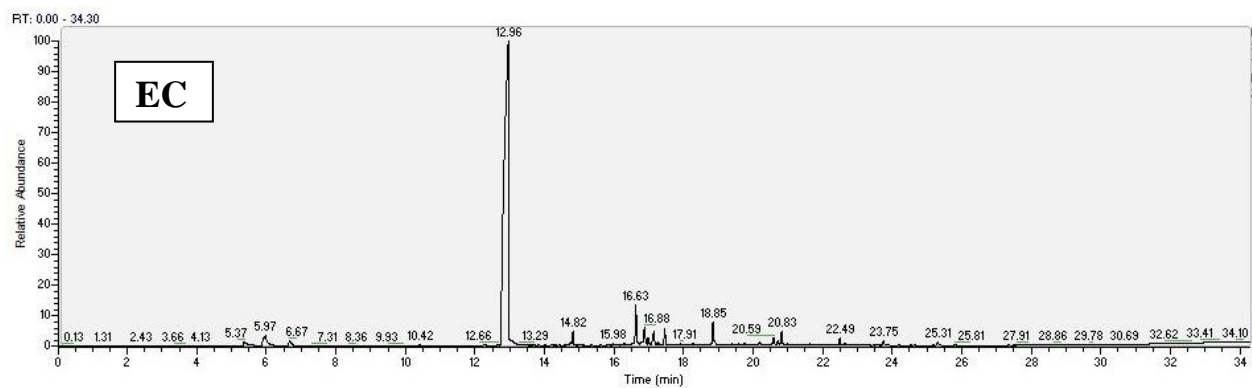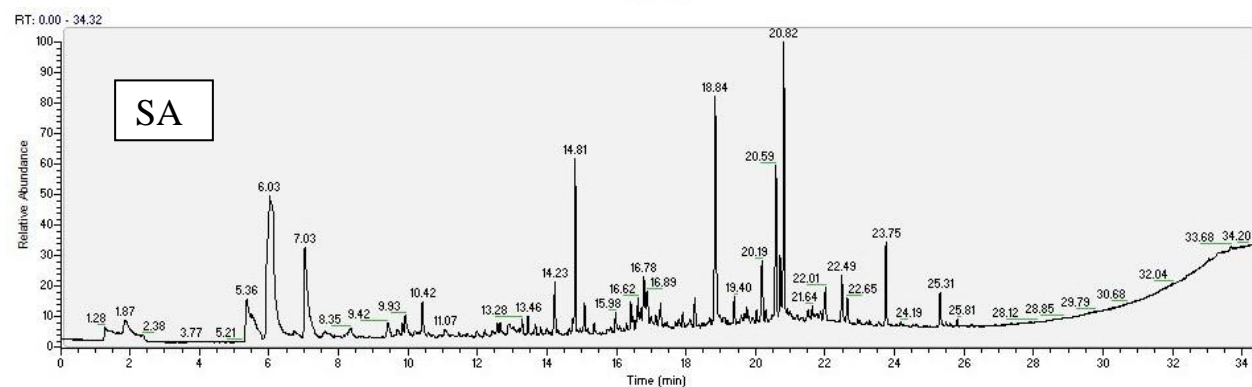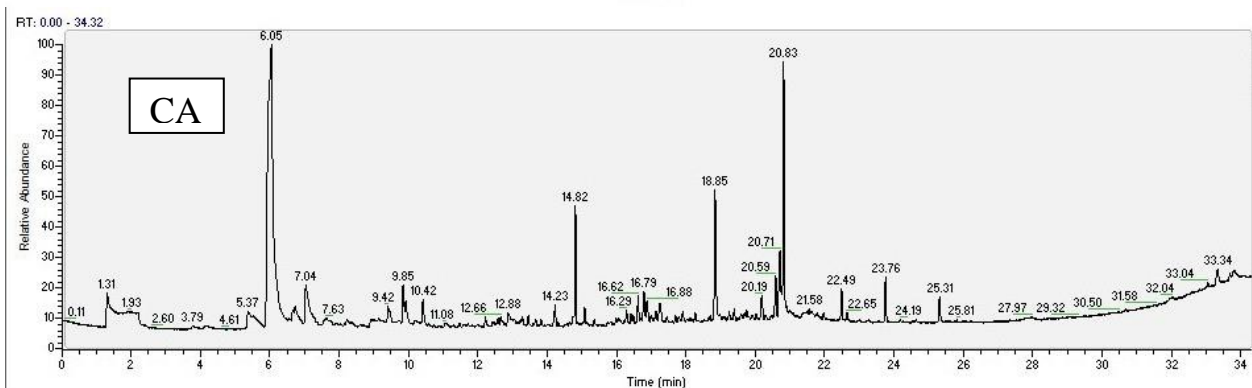

Supplement: Supplementary file 1 [file f1000research-6-14954-s0000.tgz › 9abaaadb-b5cd-413d-809f-bd94520cefd4.pdf]

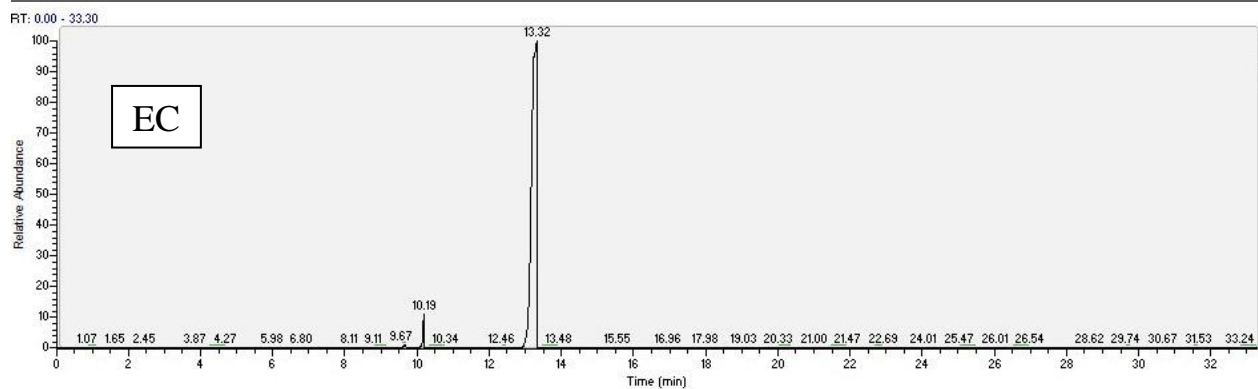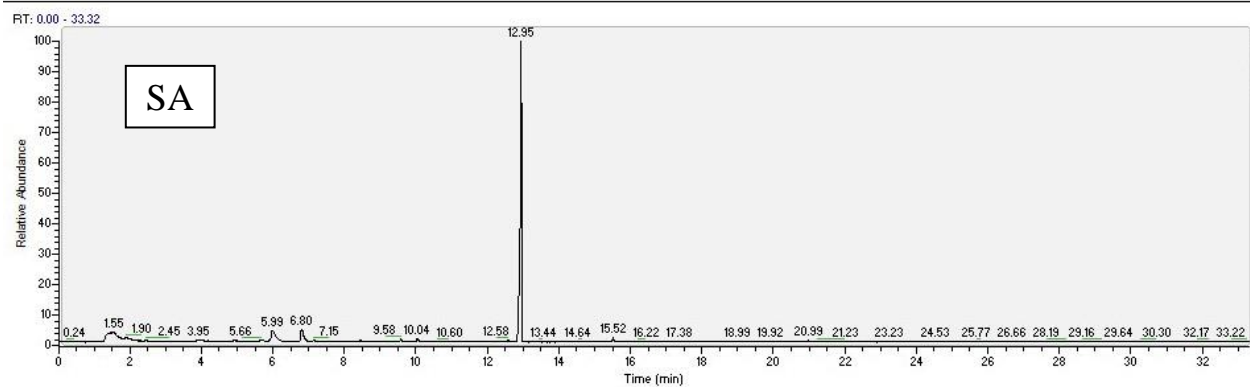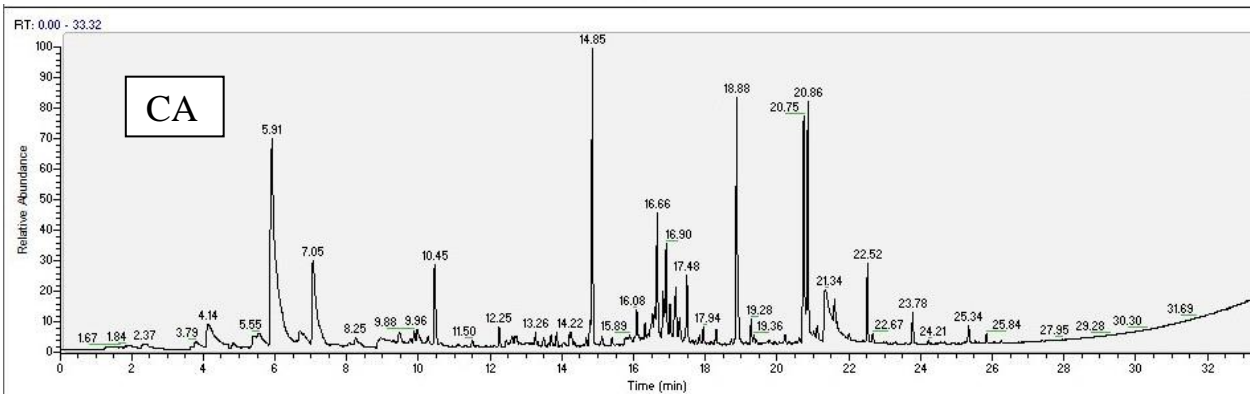

Supplement: Supplementary file 2 [file f1000research-6-14954-s0001.tgz › f10edfbb-ef2d-4d0b-a35b-c2bd6124b424.pdf]

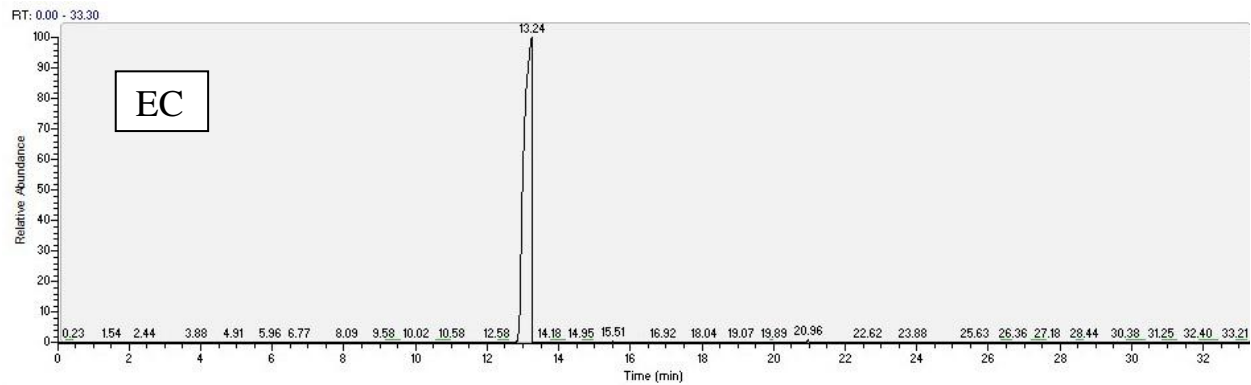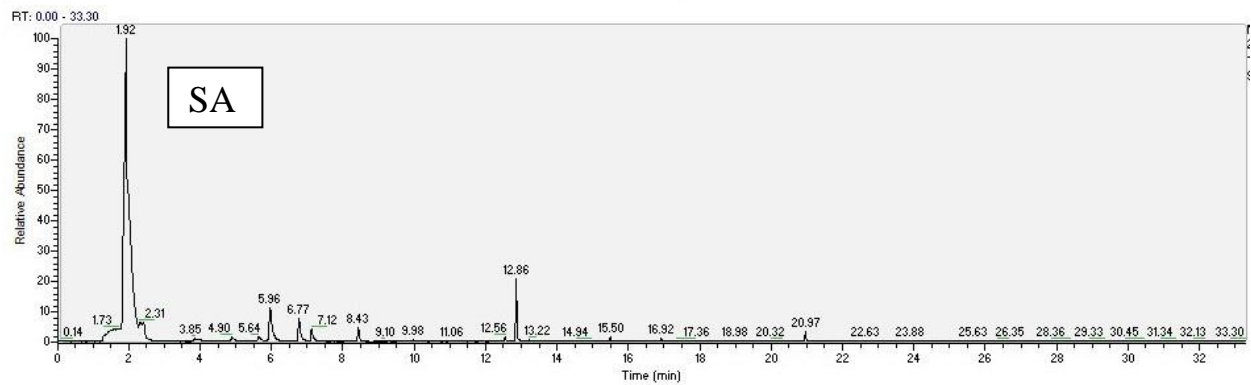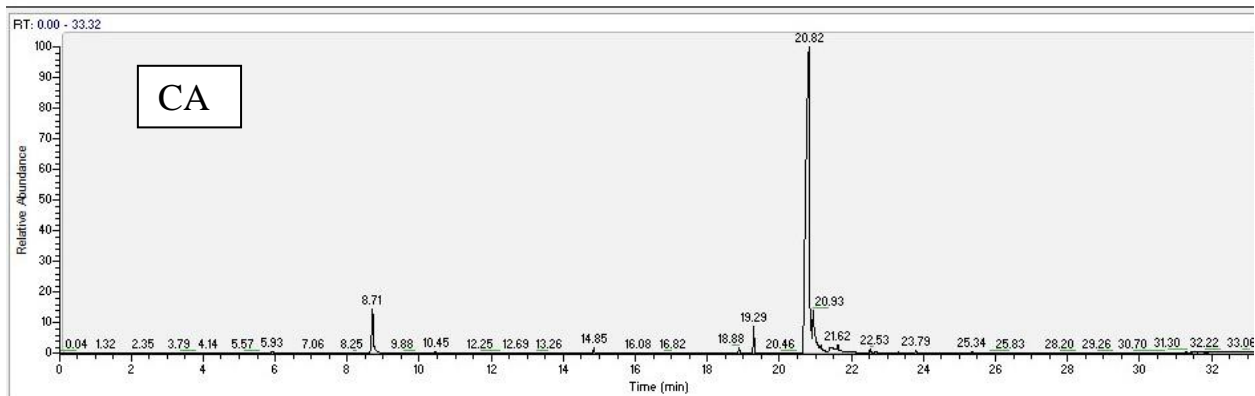

Supplement: Supplementary file 3 [file f1000research-6-14954-s0002.tgz › 2074ad66-4503-4854-b027-2fb74b4debbb.pdf]

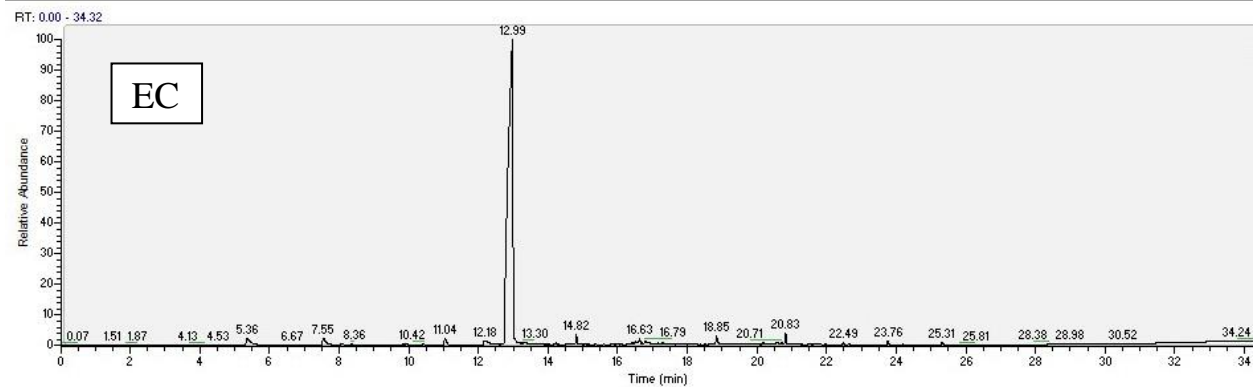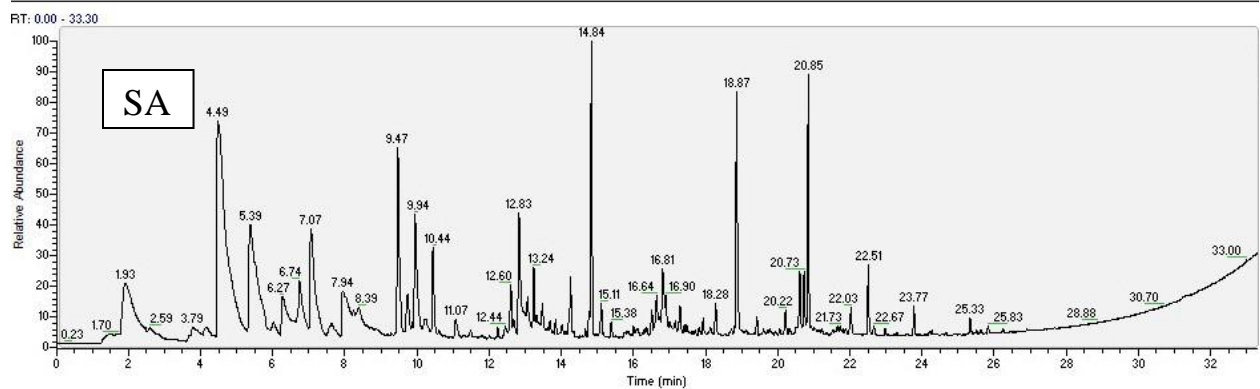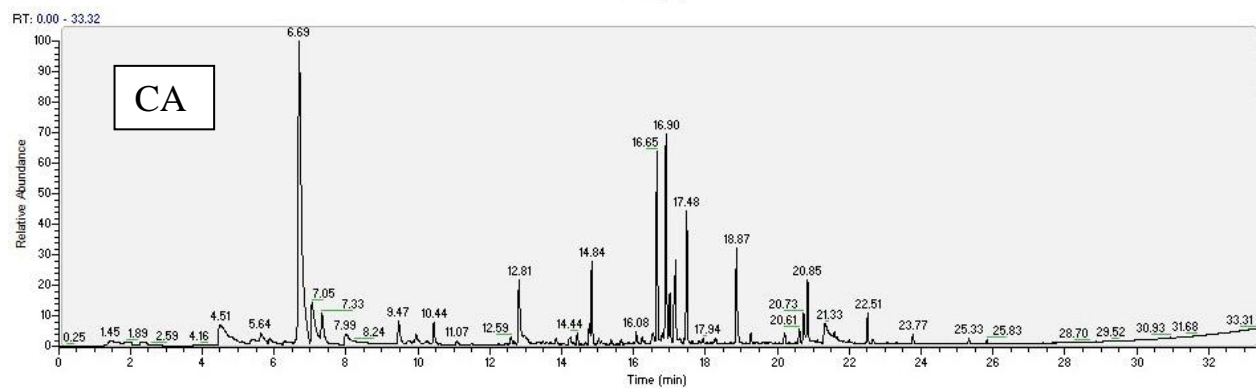

Supplement: Supplementary file 4 [file f1000research-6-14954-s0003.tgz › f62be6cb-d7d3-495b-a35b-70433750c0b5.pdf]

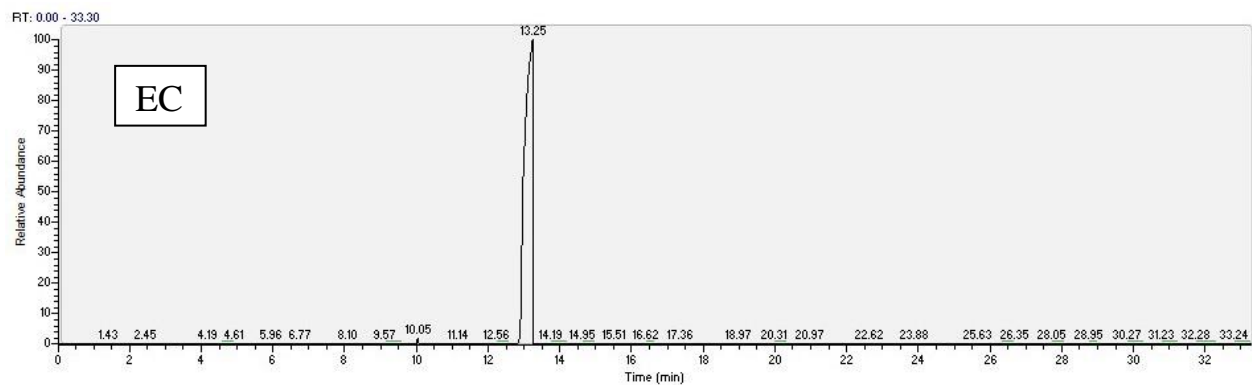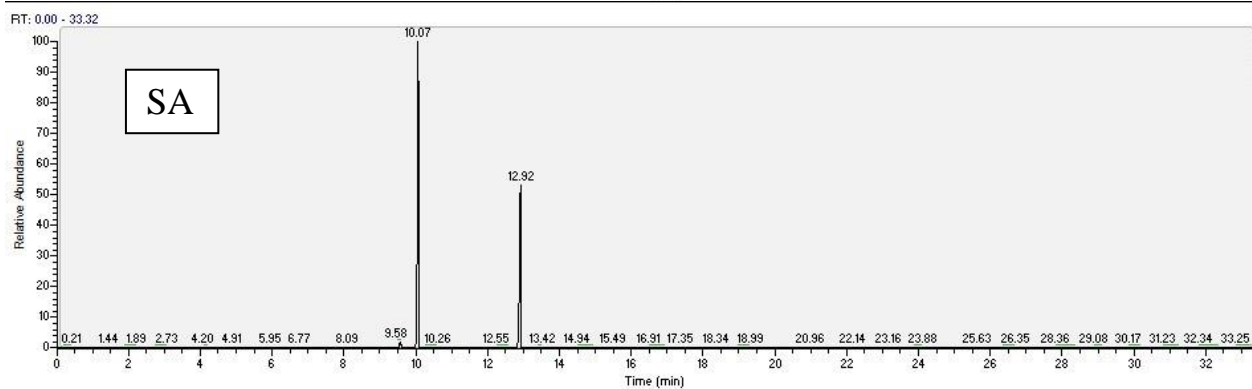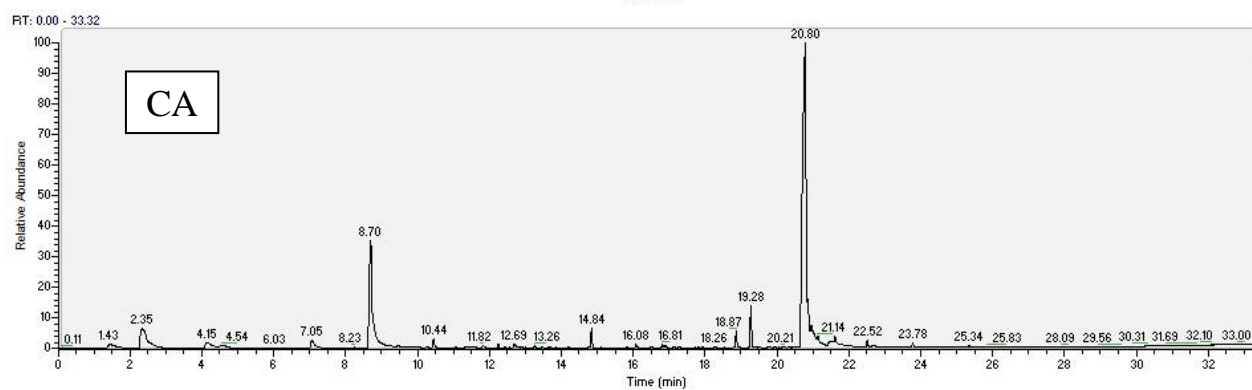

Supplement: Supplementary file 5 [file f1000research-6-14954-s0004.tgz › 0dedf5f8-1ca8-4e17-a6c2-55aa7be993a1.pdf]
