# Supplementary material for: Initial study of three different pathogenic microorganisms by gas chromatography-mass spectrometry
Source: F1000Res. 2018 Jan 18;6:1415. Originally published 2017 Aug 10. [Version 3] doi: 10.12688/f1000research.12003.3 (PMC5760968; doi:10.12688/f1000research.12003.3)
Supplement: Peer review history

# Peer review: Referee response for version 3

**DOI:** 10.5256/f1000research.14954.r29971

**Published:** 2018-01-31

**Authors:** Amy Scott-Thomas

**Affiliations:** 1 University of Otago, University of Otago, New Zealand

I have read this submission. I believe that I have an appropriate level of expertise to confirm that it is of an acceptable scientific standard.

# Peer review: Referee response for version 2

**DOI:** 10.5256/f1000research.14519.r28936

**Published:** 2018-01-10

**Authors:** Paul Brinkman

**Affiliations:** 1 Department of Respiratory Medicine, Academic Medical Center, Amsterdam, Netherlands

I would like to thank the authors for their response. Although most questions are answered, one question remains open to me:

 - Considering they have perfomed their tests in triplicate, which statistical test did the authors use to compare outcomes between and within blank and actual samples? 

I have read this submission. I believe that I have an appropriate level of expertise to confirm that it is of an acceptable scientific standard.

# Peer review: Referee response for version 2

**DOI:** 10.5256/f1000research.14519.r28937

**Published:** 2018-01-08

**Authors:** Norman Ratcliffe

**Affiliations:** 1 Institute of Biosensor Technology, University of the West of England, Bristol, UK

The paper shows some interesting preliminary results. The authors should be complimented for their good English. The works cries out for more repeats and the authors appreciate this. Within the abstract, and in the text throughout, chemical names should not have capital letters, more importantly some of the compound names need checking, particularly 1,3-heptadiene-3-yne which is in the abstract, it cannot be right.

 Also check in tables, spelling and also are you sure:

 1,2-Benzene dicarboxulic acid, this should be 1,2-benzenedicarboxylic acid

 1,3-Butadiyene, do you mean 1,3-butadiene?

 Likewise 1,2-Butadiyene ?

 1,3-Heptadiene-3-yne? Can’t be

 Dibutyl phatalate should be phthalate,  also its other name is **1,2-Benzenedicarboxylic acid, dibutyl ester. **Best to be consistent with names, I only draw attention to this  case as an example, because 1,2-benzene dicarboxylic acid has been stated to be found, and when they have the same naming, its brings out the thought that the acid may have come from the ester (I say this, rather than the other way around as the butyl ester is a common plasticizer, and can be a contaminant).

 Longifolrne spelling! And it appears just below, they are the same? Do they have the same rts?

 Longifolene

 Phatalic acid, butyl ester, spelling

 Sesquiphellandrene  and beta- Sesquiphellandrene, are both listed are they the same?

 Table 2,3

 Check as above.

 There are some compounds, which may be speculatively  assigned, eg 4-t-butyl-2-(1-methyl-2- nitroethyl)cyclohexane? Are the authors really sure

 Supplementary : E. coli:5.5-Dodecadinyl-1, 12-diol, can’t be right, check, plus check names of chemicals as above

I have read this submission. I believe that I have an appropriate level of expertise to confirm that it is of an acceptable scientific standard.

# Peer review: Referee response for version 1

**DOI:** 10.5256/f1000research.12982.r27814

**Published:** 2017-11-27

**Authors:** Norman Ratcliffe

**Affiliations:** 1 Institute of Biosensor Technology, University of the West of England, Bristol, UK

The researchers tackle an area of significant interest, rapid determination of bacterial species, especially associated with life threatening illness, using a relatively new approach, that of VOC analyses. In general the paper reads well.

 The analytical method for VOC analyses is ok. Could the authors confirm whether the cfu/ml count is approx. for the different species at the same time points to enable a good comparison of VOCs to be made.  One major weakness to the work is that only one analysis for each species at one time point and media was undertaken, the literature gives examples of several analyses being undertaken for similar studies. The title is too ambitious, rather than diagnoses being purported, maybe an” initial study of…” would be more accurate.

 The authors could comment on how their research would ultimately fit into a clinical test, if the VOCs were to be analysed in breath, would the same volatile profile be expected?

 In the text it was stated that “Extensive studies were also performed by a phytochemist to determine if the compounds were organic” , some explanation would be good as to what this means.

 Some other matters, VOC abbreviation used twice in the abstract, also in quite a few places the chemical names have capital letters and are misspelt and merged with another word.

 In Results, what is uncommon determined by, some more discussion on stats…

I have read this submission. I believe that I have an appropriate level of expertise to confirm that it is of an acceptable scientific standard, however I have significant reservations, as outlined above.

# Peer review: Referee response for version 1

**DOI:** 10.5256/f1000research.12982.r25909

**Published:** 2017-11-15

**Authors:** Amy Scott-Thomas

**Affiliations:** 1 University of Otago, University of Otago, New Zealand

This paper looks at the detection of VOC's produced by Escherischia coli, Staphylococcus aureus and Candida albicans using solid phase micro-extraction coupled with gas chromatography / mass spectrometry. 

 Its seems as though the group only ran this experiment once so while they have pointed out the detection of specific volatiles at differing time points this needs to be repeated to ensure that it is indeed a true and repeatable release from the micro-organisms.

 The work is outlined clearly and the results listed comprehensively however there has been no indepth discussion surrounding why these volatiles have been produced in vitro, would they be produced in vivo nor an extensive list of references to back this up. 

 While they state Indole as the most important volatile produced by E.coli they do not go into any detail regarding how this would relate to an actual breath test since indole can be released by mouth flora. Also as indole is produced from tryptophan discussion around the levels of tryptophan in the lung and therefore its actual availability to E.coli growing in the lung is pertinent. Growth in a minimal media with varying tryptophan levels would give more insight.

 It would have also been beneficial to look at the effect of co-culturing these micro-organisms as this may alter the release of the detected VOC's and initiated the production of others.

I have read this submission. I believe that I have an appropriate level of expertise to confirm that it is of an acceptable scientific standard, however I have significant reservations, as outlined above.

# Peer review: Referee response for version 1

**DOI:** 10.5256/f1000research.12982.r25394

**Published:** 2017-09-08

**Authors:** Paul Brinkman

**Affiliations:** 1 Department of Respiratory Medicine, Academic Medical Center, Amsterdam, Netherlands

General Comments:

 This study aimed to identify typical volatile organic compounds (VOCs) produced by *Staphylococcus aureus*, *Escherichia coli *and *Candida albicans* by extraction of cultured bacterial strain headspace using solid phase microextraction (SPME) fibers analyzed by Gas Chromatography-Mass Spectrometry (GC-MS). Although the aim of study is obvious and very relevant data is shown, some extension on utilized statistics is needed. Additionally, a replication and/or validation of findings is crucial. The authors do show that some of the VOCs, e.g. Indole, are revealed at multiple time points, but confirmation of observations by repetition of experiments is recommended.

 Major Comments:

- To avoid possible false discoveries a repetition of experiments is recommended.
- Which (statistical) methods were used in order to compare blank vs. actual samples?  
- Which criteria were applied in order to confirm a ‘match’ between detected volatiles and the NIST library?
- The authors nicely show the overlap and/or discrepancy between their findings and current available literature, but what about the interpretation of findings? Can the authors extend and/or speculate about possible biological mechanisms behind the revealed VOCs

Minor comment:

  

- Please consider as reference: Neerincx *et al*; Identification of Pseudomonas aeruginosa and Aspergillus fumigatus mono- and co-cultures based on volatile biomarker combinations; J Breath Res. 2016 Jan 29;10(1):016002.

I have read this submission. I believe that I have an appropriate level of expertise to confirm that it is of an acceptable scientific standard, however I have significant reservations, as outlined above.

## References

[1] NeerincxAHGeurtsBPHabetsMFBooijJAvan LoonJJansenJJBuydensLMvan IngenJMoutonJWHarrenFJWeversRAMerkusPJCristescuSMKluijtmansLA: Identification of Pseudomonas aeruginosa and Aspergillus fumigatus mono- and co-cultures based on volatile biomarker combinations. *J Breath Res*.2016;10(1) : 10.1088/1752-7155/10/1/016002 016002 10.1088/1752-7155/10/1/016002 26824272
